# Patient-reported quality of life outcomes for children with serious congenital heart defects

**DOI:** 10.1136/archdischild-2013-305130

**Published:** 2014-01-09

**Authors:** Rachel L Knowles, Thomas Day, Angie Wade, Catherine Bull, Christopher Wren, Carol Dezateux, On behalf of the UK Collaborative Study of Congenital Heart Defects (UKCSCHD)

**Affiliations:** 1 MRC Centre of Epidemiology for Child Health, Centre of Paediatric Epidemiology and Biostatistics, UCL Institute of Child Health, University College London, London, UK; 2 Cardiac Unit, Great Ormond Street Hospital for Children NHS Trust, London, UK; 3 Department of Paediatric Cardiology, Freeman Hospital, Newcastle-upon-Tyne, UK

## Abstract

**Objective:**

To compare patient-reported, health-related quality of life (QoL) for children with serious congenital heart defects (CHDs) and unaffected classmates and to investigate the demographic and clinical factors influencing QoL.

**Design:**

Retrospective cohort study.

**Setting:**

UK National Health Service.

**Patients:**

UK-wide cohort of children with serious CHDs aged 10–14 years requiring cardiac intervention in the first year of life in one of 17 UK paediatric cardiac surgical centres operating during 1992–1995. A comparison group of classmates of similar age and sex was recruited.

**Main outcome measures:**

Child self-report of health-related QoL scores (Pediatric Quality of Life Inventory, PedsQL) and parental report of schooling and social activities.

**Results:**

Questionnaires were completed by 477 children with CHDs (56% boys; mean age 12.1 (SD 1.0) years) and 464 classmates (55%; 12.0 (SD 1.1) years). Children with CHDs rated QoL significantly lower than classmates (CHDs*:* median 78.3 (IQR 65.0–88.6); classmates: 88.0 (80.2–94.6)) and scored lower on physical (CHDs: 84.4; classmates: 93.8; difference 9.4 (7.8 to 10.9)) and psychosocial functioning subscales (CHDs: 76.7, classmates: 85.0; difference 8.3 (6.0 to 10.6)). Cardiac interventions, school absence, regular medications and non-cardiac comorbidities were independently associated with reduced QoL. Participation in sport positively influenced QoL and was associated with higher psychosocial functioning scores.

**Conclusions:**

Children with serious CHDs experience lower QoL than unaffected classmates. This appears related to the burden of clinical intervention rather than underlying cardiac diagnosis. Participation in sports activities is positively associated with increased emotional well-being. Child self-report measures of QoL would be a valuable addition to clinical outcome audit in this age group.

**What is already known**

- Health, educational and quality of life (QoL) outcomes have increasing relevance to children with serious congenital heart defects (CHDs), who can now expect to survive into adulthood.
- Studies comparing health-related QoL outcomes of school-age children with serious CHDs and their classmate peers are lacking.
- Children's own self-reported views are important outcome measures as the child's perspective often differs from parents.

**What this study adds**

- Ten to 14-year-olds with serious congenital heart defect (CHDs) report significantly lower health-related quality of life than unaffected classmates.
- This reduction is related to the burden of clinical intervention and on-going care rather than cardiac diagnosis; sports participation offers positive benefit to psychosocial functioning.
- Collection of child-reported outcomes for CHDs is practicable and its inclusion in routine national clinical outcome monitoring and audit should be considered.

## Introduction

Increasing numbers of children operated in infancy for serious congenital heart defects (CHDs) are surviving through childhood and into adulthood.^1^ As mortality falls for these children, broader health outcomes of significance to children and their families, such as health-related quality of life (QoL) and the capacity for social and educational participation attain greater importance.^2^ Over a decade ago, the Bristol Inquiry^3^ report into the care of children undergoing cardiac surgery highlighted the lack of long-term outcome data for children with CHDs and underlined the need for a national monitoring system. Although short-term cardiac surgical outcomes for UK children are now comprehensively collected by the Central Cardiac Audit Database and published through the NICOR-Congenital Heart Disease Portal,^4^ the quality of long-term survival at a national level is not routinely captured.

Patient-reported experiences and outcomes are central to quality improvements within the National Health Service (NHS)^5^ and are increasingly being advocated for monitoring individual clinical care,^6^ ^7^ which has led to an expansion in instruments designed to measure well-being, QoL and healthcare experience. Patient-reported outcome measures (PROMS) ascertain the patient's own assessment of their health, functional status and QoL. The application of PROMS in clinical practice and outcomes research is pertinent to all chronic childhood disorders, of which CHDs are a key example, as outcomes will vary across the lifetime influenced by children's adaptation to their changing environment and particularly the transition to adulthood. While age-adapted questionnaires specifically suited to self-reporting of health outcomes by children have multiplied, there remains a reliance on proxy reporting by parents. Although parental perspectives are useful, QoL and patient experience are subjective concepts. Consequently children's own views on their health and well-being should be assessed. Moreover, evidence suggests that children's views are reliable and can differ greatly from the views of their parents or education and health professionals.^8^

QoL measures focus on daily life experiences and outcomes during childhood and adolescence and facilitate the development of interventions to support families and promote resilience, or positive adaptation, in long-term survivors with chronic disorders.^9^ A limited number of multidimensional patient self-report instruments have been validated to explore the child's perspective on health and well-being in paediatric cardiac populations, including the impact of a CHD on current lifestyle, past experience and future expectations.^2^ ^10–12^ The Pediatric Quality of Life Inventory (PedsQL 4.0)^13^ is a widely employed generic QoL instrument for children aged 2–18 years; the questionnaire may be self-completed by children aged 5 years or over.

Our aims were to estimate and compare QoL scores reported using the PedsQL 4.0 by a UK-wide cohort of school-age children with serious CHDs, with a comparison group comprised of their unaffected classmates and to investigate the factors that influence self-reported outcomes for children living with chronic or congenital conditions.

## Methods

The UK Collaborative Study of CHDs (UKCSCHD) is a multicentre prospective study of almost 4000 children, born 1992–1995 with serious CHDs requiring intervention within the first year of life and involving all 17 UK paediatric cardiac centres operating at that time. The cohort excluded children with minor defects not requiring intervention,^14^ but included around one-third of all children with CHDs (2/1000 live births). Information, including sex, cardiac diagnosis and cardiac procedures, was obtained from individual case notes review. Each child was assigned a primary cardiac diagnosis using Wren's hierarchy^14^ and a diagnosis-derived cardiac prognostic severity (CPS) score, adapted from Lane^15^ (see online supplementary table S1). Research ethics approval (Trent MREC 04/4/017) was given for local cardiologists to contact surviving children with an invitation to participate in the postal questionnaire follow-up of QoL, health and educational outcomes.

**Figure 1:**
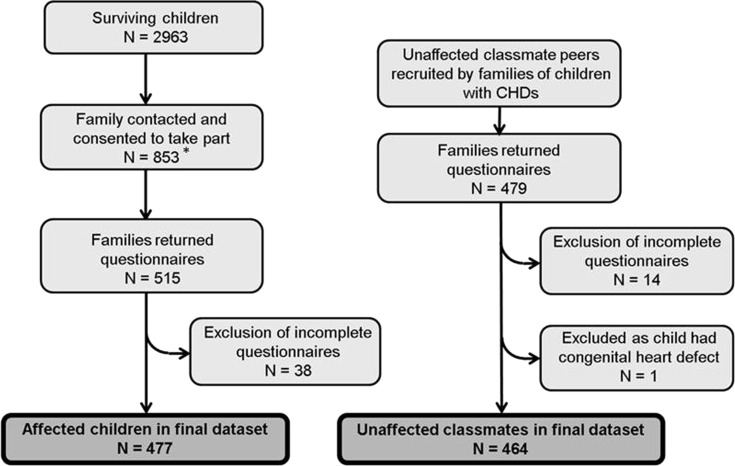
Flow diagram of recruitment and response to questionnaires. *Contact with families by researchers was dependent on the local clinician obtaining consent to contact from each child's general practitioner (GP). If a GP did not provide written consent or did not respond, questionnaires were not sent to the family. This procedure was mandated by the research ethics committee in order to protect patient confidentiality and the central study team did not receive details of local response rates. On this basis of the London mailing, which did involve members of the study team, we estimate that only 70% of survivors were sent an invitation to participate, but a proportion of these were addressed incorrectly and/or returned unopened. Throughout the UK, 853 families agreed to receive a questionnaire and 515 (60.3%) of these were returned completed.

During 2004–2007, local collaborating cardiologists attempted to contact 2963 surviving children, then aged 10–14 years (figure 1). Due to restrictions imposed by ethics and governance approvals,^16^ families could only be contacted by the local clinical care team after approval by the child's cardiologist and general practitioner; we estimate that around 70% of eligible children received an invitation to participate. Of 853 children who agreed to take part, 515 (60.3%) returned a questionnaire; 38 were subsequently excluded due to incomplete responses (figure 1). In addition, parents returned a parent questionnaire for 19 children who did not return the child questionnaire; 12 of these children were reported by parents to have significant learning difficulties which may account for non-completion. Participants were asked to recruit a comparison group by giving additional questionnaires to their two classmates closest in age and of the same sex. We received questionnaires from 479 classmates; 15 were excluded as incomplete/ineligible.

Each child completed the PedsQL 4.0 questionnaire (UK English version). Items are scored on a Likert scale from 0 (never a problem) to 4 (almost always a problem) then transformed to a 0–100 scale to provide physical functioning, psychosocial (school, social and emotional) functioning and summary scores; higher scores represented a better QoL.^13^ Scores were not calculated if more than half the scale items were incomplete (physical scale incomplete (n=3); emotional scale incomplete (n=1)). We estimated the minimal clinically important difference (MCID), defined as the minimum score increase on a QoL scale for a treatment to be considered of patient benefit and compared this with the MCID of 4.4 points attributed to the unadjusted Child Report PedsQL Summary Scale.^13^

Parents (including carers or guardians acting in a parental role) completed a questionnaire, providing information about their employment, education, family and their child's health, schooling and daily activities. Parental employment was full time (1.0) or part-time (0.5) for each parent, then summed. Parents reported regular medications (classified as cardiac or non-cardiac), vision, hearing or speech problems, special educational needs provision, school absences and participation in sporting and social activities for their child. Parents were asked whether they considered their child to have a long-standing non-cardiac illness, and if so, whether this limited their child's activities.^17^

### Statistical analysis

Participant characteristics were examined for response bias. As PedsQL scores were not normally distributed, median scores were compared between groups and 95% CIs for the difference in medians estimated.^18^ To explore factors influencing outcome, we developed univariable and multivariable regression models, using Generalised Additive Models for Location, Scale and Shape (GAMLSS) based on the Sinh-Arcsinh (SHASH) distribution, to take account of the non-normal outcome distribution.^19^ ^20^ A forward variable-selection approach was used and variables retained if they improved goodness-of-fit, based on the Akaike information criterion. We assessed the need for a multilevel model to account for correlation within cardiac centres and case–control clusters; no evidence of correlation within cardiac centres was found and we adjusted for clustering of cases and controls by including three levels of school-type factors (mainstream school, mainstream school with learning support or special school/unit) in regression models.

Sensitivity analyses explored the effect of excluding children who did not recruit classmates, as these children were more likely to attend special schools. Statistical analyses were performed in R V.12.2.1 (R Foundation for Statistical Computing, Austria).

## Results

Data from 477 affected children (268 boys (56%); mean age 12.1 (SD 1.0) years) and 464 classmates (255 boys (55%); mean age 12.0 (SD 1.1) years) were analysed. Characteristics of children with CHDs were compared with non-responding survivors (n=2486) in the UKCSCHD cohort (see online supplementary table S1). Children returning questionnaires were representative of all primary cardiac diagnoses, although children with more severe CPS scores (palliated CHDs) appeared more likely to return questionnaires. Characteristics of affected children and classmates were compared (table 1); children with CHDs were on average 200 g lighter at birth, more likely to take regular medications, have associated health problems and health-related absences from school and participated less frequently in sport and social activities.

**Table 1:** Characteristics of affected children and their classmates participating in the study

	Children with CHDs N=477		Unaffected classmates N=464		
	N (%)	Missing (N (%))	N (%)	Missing (N (%))	p Value for difference
*Individual factors*					
Male	268 (56)	0	255 (55)	0	0.63
Age (years)	12.1 (1.0)*	0	12.0 (1.1)*	0	0.33
Ethnicity		4 (1)		7 (2)	0.24
White	443 (93)		420 (90)		
Non-white	30 (6)		37 (8)		
Birth weight (g)	3228 (673.5)*	16	3443 (603.0)*	15	<0.001
*Parent*† *and family factors*					
Parental education level		0		0	0.14
None/General Certificate of Secondary Education	168 (35)		142 (31)		
A level	140 (29)		129 (28)		
Degree	169 (36)		193 (41)		
Number of full-time equivalent working parents†	1.2 (0.58)*	0	1.3 (0.56)*	0	0.19
Number of siblings at home	2.0 [2.0–2.0]‡	0	2.0 [2.0,2.0]‡	0	0.44
Two parents† at home at birth	452 (95)	0	444 (96)	2	0.22
Two parents† at home now	385 (81)	0	364 (78)	1	0.43
Mother's age at child's birth (years)	29.6 (5.1)*	6	29.8 (4.8)*	8	0.53
Father's age at child's birth (years)	32.1 (6.1)*	11	32.0 (5.9)*	11	0.86
*Comorbidities*					
Non-cardiac long-standing illness		20		13	<0.001
Yes, not limiting	66 (14)		48 (10)		
Yes, limiting	111 (23)		31 (7)		
Uses regular non-cardiac medications	170 (36)	3	40 (9)	7	<0.001
Problems with vision	164 (34)	14	109 (23)	12	<0.001
Problems with hearing	124 (26)	5	69 (10)	10	<0.001
Problems with speech	70 (15)	9	13 (3)	7	<0.001
*School and daily life activities*					
Type of schooling		0		0	<0.001
Mainstream school	326 (68)		418 (90)		
Mainstream with assistance	112 (23)		40 (9)		
Special school/unit	39 (8)		6 (1)		
School absence in last year		4		5	<0.001
Never	105 (22)		148 (32)		
<1 week	193 (40)		227 (49)		
1–2 weeks	102 (21)		56 (12)		
2 weeks–1 month	46 (10)		20 (4)		
>1 month	28 (6)		8 (2)		
Frequency of sport§	1.0 [0.5–3.0]‡	0	2.0 (1.0–3.0)‡	0	<0.001
Frequency of social activities¶	5.0 [2.5–6.5]‡	0	6.0 (4.0–7.2)‡	0	<0.001
*Cardiac factors*					
No cardiac disorder	0		464 (100)	0	N/A
Cardiac prognostic severity (CPS)		1 (<1)	N/A		*** ***
Curative	102 (21)				*** ***
Corrective	272 (57)				*** ***
Palliative	103 (22)				*** ***
Number of cardiac interventions	2.0 [1.0–3.0]‡	52	0	0	N/A

*Mean (SD).

†Includes carers/guardians.

‡Median [IQR] based on parent questionnaire (parents reported all interventions where hospital case notes were incomplete and missing data reflects where parents could not provide data).

§Number of occasions child takes part in a sporting activity (not school PE) per week.

¶Number of occasions outside of school hours that a child takes part in sport, plays with friends or attends non-sport clubs per week.

CHD, congenital heart defect.

**Figure 2:**
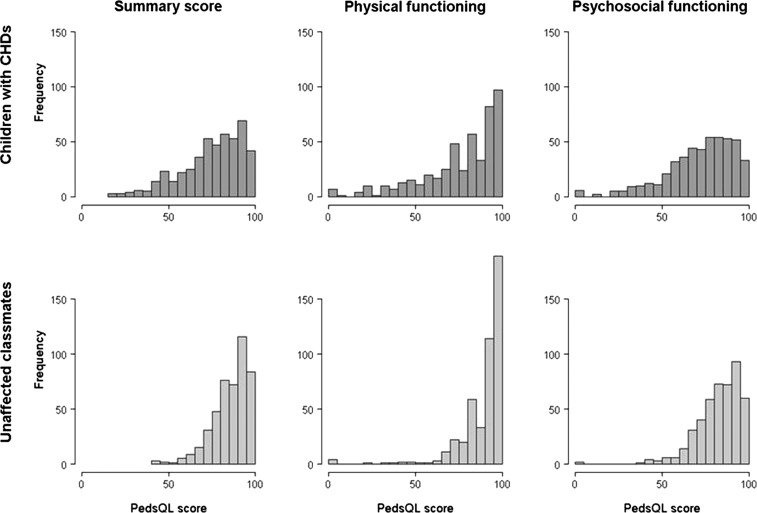
Unadjusted median PedsQL scores for children with CHDs (n=477) compared with unaffected classmates (n=464).

Unadjusted median PedsQL physical functioning, psychosocial functioning and summary scores for children with CHDs and unaffected classmates differed significantly (figure 2). The unadjusted median summary score was 78.3 (IQR 65.0–88.6) for the affected children, compared to 88.0 (80.2–94.6) for unaffected classmates (difference 9.8 (95% CI 7.1 to 12.4)). Unadjusted median physical and psychosocial functioning scores for children with CHDs were (84.4 (65.6–93.8) and 76.7 (62.5–86.7)), respectively; difference 9.4 (95% CI 7.8 to 10.9)) and significantly lower than for unaffected classmates (93.8 (84.0–100.0) and 85.0 (76.7–93.3), respectively; difference 8.3 (95% CI 6.0 to 10.6)).

In univariable regression models (see online supplementary table S2), factors significantly associated with lower scores included worse CPS score, increasing number of cardiac interventions, long-standing limiting non-cardiac illness, regular medications, longer school absence, and vision or speech problems. Hearing difficulties were associated with worse psychosocial functioning and summary scores only. Increasing frequency of sporting and social activities was associated with higher PedsQL scores, and higher psychosocial functioning and summary scores were associated with both parents living in the family home and parents in employment.

In multivariable models (table 2), adjusting for school-type and sociodemographic factors such as parent education or employment, having a CHD remained an independent predictor of significantly worse summary, physical and psychosocial functioning scores. Non-cardiac comorbidities, indicated by limiting long-standing illness and regular medications, were also independently associated with poorer outcomes. Visual or hearing difficulties were associated with lower summary scores only, whereas school absence was associated with poorer summary and psychosocial, but not physical, functioning scores. Increased frequency of sports participation remained associated with better summary and psychosocial, but not physical, functioning scores (table 3A). Individual child age, sex or ethnicity, and parental educational or employment, did not independently influence self-reported QoL for children in our study. These findings persisted in sensitivity analyses.

**Table 2:** Multivariable models to investigate factors associated with PedsQL outcome scores

	PedsQL summary score			Physical functioning			Psychosocial functioning		
PedsQL	Est.	SE	p Value	Est.	SE	p Value	Est.	SE	p Value
*Individual factors*									
Presence of CHD	−2.58	0.77	<0.001	−1.70	0.57	0.003	−2.35	0.93	0.01
Female	−0.29	0.77	0.71	−0.91	0.57	0.11	0.27	0.87	0.76
Age	−0.30	0.38	0.42	−0.42	0.29	0.15	−0.12	0.44	0.78
White	−0.12	1.70	0.95	0.41	1.21	0.73	0.03	2.07	0.99
Birth weight (per kg)	−0.32	0.57	0.58	−0.66	0.37	0.07	0.016	0.71	0.98
*Parent*† *and family factors*									
Parental education									
None/General Certificate of Secondary Education	*ref*	0.94	0.73	*ref*	0.79	0.45	*ref*	1.06	0.96
A level	0.33	0.89	0.83	0.59	0.72	0.56	−0.05	1.03	0.77
Degree	0.19			0.42			0.30		
Number of full-time equivalent working parents†	−0.09	0.81	0.91	−0.24	0.72	0.74	−0.35	0.92	0.71
Number of siblings at home	6.09	8.16	0.46	7.12	5.80	0.21	3.44	8.98	0.70
Two parents† at home now	−4.63	8.20	0.57	−7.38	5.71	0.20	−1.23	9.05	0.89
Two parents† at birth	−1.23	1.75	0.48	−0.46	1.61	0.78	−2.37	2.12	0.27
Mother's age at birth	0.09	0.11	0.42	−0.07	0.09	0.44	0.12	0.13	0.33
Father's age at birth	−0.01	0.10	0.94	0.04	0.07	0.53	−0.01	0.11	0.91
*Comorbidities*									
Non-cardiac long-standing illness									
None	*ref*	1.17	0.88	*ref*	0.99	0.52	*ref*	1.35	0.57
Yes, not limiting	−0.17	1.04	<0.001	−0.64	1.14	<0.001	−0.76	1.19	<0.001
Yes, limiting	−5.71			−2.25			−6.20		
Uses regular medications	−3.74	0.98	<0.001	−2.45	0.91	0.008	−2.90	1.12	0.001
Problems with vision	−1.90	0.85	0.03	−0.68	0.68	0.32	−1.85	0.98	0.06
Problems with hearing	−2.32	0.97	0.02	−1.00	0.74	0.18	−2.05	1.11	0.06
Problems with speech	2.04	1.58	0.20	1.91	1.57	0.24	−0.16	1.67	0.92

Adjusted for school type, that is, mainstream school, mainstream school with learning support or special school/unit.

†Includes carers/guardians.

CHD, congenital heart defect.

**Table 3:** Multivariable models to investigate the additional effect on PedsQL outcome scores of daily life activities and cardiac severity (children with CHDs only)

	PedsQL summary score			Physical functioning			Psychosocial functioning		
PedsQL	Est.	SE	p Value	Est.	SE	p Value	Est.	SE	p Value
*A: Daily life activities*‡									
School absence in last year									
Never	*ref*	1.07	0.20	*ref*	0.82	0.46	*ref*	1.00	0.07
<1 week	−1.37	1.23	<0.001	−0.60	0.95	0.03	−1.79	1.40	0.002
1–2 weeks	−4.46	1.65		−2.12	1.21	0.003	−4.43	1.84	0.004
2 weeks–1 month	−5.29	1.81	0.001	−3.54	1.56	0.23	−5.33	2.69	<0.001
>1 month	−10.12		<0.001	−1.87			−10.38		
Frequency of sport*	0.86	0.40	0.003	0.21	0.28	0.56	0.89	0.41	0.03
Frequency of social activities†	−0.08	0.20	0.68	0.05	1.58	0.76	−0.18	0.22	0.40
*B: Cardiac severity (children with CHDs only)§*									
Cardiac factors									
Cardiac Prognostic Severity (CPS)									
Curative	*ref*	1.55	0.45	*ref*	1.25	0.68	*ref*	1.81	0.35
Corrective	−1.17	1.35	0.91	0.52	1.11	0.93	−1.8	1.57	0.94
Palliative	0.14			0.09			0.11		
Number of cardiac interventions	−0.74	0.16	<0.001	−0.56	0.21	0.007	−0.69	0.19	<0.001

*Number of occasions child takes part in a sporting activity (not school PE) per week.

†Number of occasions outside of school hours that a child takes part in sport, plays with friends or attends non-sport clubs per week.

‡Adjusted for school type, individual factors, parent and family factors and comorbidities.

§Adjusted for school type, individual factors, parent and family factors, comorbidities and activities of daily life.

Est., estimate.

For children with CHDs physical functioning, psychosocial functioning and summary scores decreased significantly as the number of cardiac interventions increased; conversely the diagnosis-based CPS score was not associated with outcome (table 3B).

## Discussion

Our study demonstrates that children with serious CHDs report significantly impaired QoL compared with their unaffected classmates, with unadjusted median scores 8–10 points lower on the summary, physical and psychosocial functioning PedsQL scales. The difference we observed between affected children and their classmates exceeds the MCID for the PedsQL summary scale and therefore has clinical relevance. Significant differences persisted after adjustment for age, sex, ethnicity and sociodemographic factors. Specific CHD diagnosis was not associated with QoL, whereas a higher cumulative burden of cardiac interventions over the lifetime had a significant negative impact. Additional factors associated with reduced QoL independently of having a CHD were non-cardiac comorbidities, specifically long-standing limiting non-cardiac illness, vision or hearing difficulties, and the need to take regular medications or time off from school for health reasons. Our findings suggest that children whose full inclusion in school and social activities is limited by a chronic disorder, whether this is cardiac or non-cardiac, experience lower subjective QoL.

Latal^2^ identified seven studies in which QoL after cardiac surgery was measured using child self-report instruments. Study findings were inconsistent, which could be related to instrument heterogeneity or variable participant selection. In studies of children with chronic conditions, worse PedsQL scores were reported by children with cardiac defects in comparison with population norms^10^ ^21–23^ or ‘healthy’ comparison groups.^24–26^ Children with more ‘severe’ CHDs rated QoL lower, although ‘severe’ was variously defined as cyanosis,^24^ or type of intervention.^10^ ^21^ Many studies involved only a single CHD diagnosis;^2^ ^23^ ^26–28^ therefore, generalisability is limited. Although methodological differences limit direct comparison with our findings, studies involving paediatric chronic disease groups have demonstrated reductions in PedsQL scores comparable with CHDs, for children with end-stage renal disease, epilepsy and cystic fibrosis,^21^ ^22^ ^29^ while children with diabetes experience less reduction in QoL.^21^

Our prospective population-based study included all UK paediatric cardiac surgical centres and is representative of children born during the 1990s with a CHD requiring intervention. Only children who had a cardiac intervention before the age of 1 year were included, nevertheless few children with complex and significant CHDs will have been excluded by this pragmatic definition of severity.^14^ ^30^ Although governance restrictions limited the questionnaire to two-thirds of survivors,^16^ we achieved 60% response rate from families and comparison of respondent and non-respondent characteristics did not indicate systematic bias. A limitation of our postal survey method is that we are unable to verify whether children completed their questionnaires without support or influence from others and, as noted above, some children with learning difficulties were unable to complete their questionnaire. Children with palliated defects appeared more likely to respond; however, these children also had more frequent outpatient visits so their contact details were more likely to be current. Over 400 classmates comprised our healthy comparison group, thus avoiding reliance on population norms and facilitating analysis of factors associated with QoL. Importantly, mean classmate PedsQL scores were similar to those previously reported for healthy children.^31^ To take account of the non-normal distribution of participant factors and outcome scores, we employed non-parametric statistical methods, including GAMLSS regression models. To our knowledge this is the first time that regression analyses have adjusted for the skewed distribution of PedsQL scores using these methods.

Children in our study were more likely to experience reduced QoL if they could not attend school or take part in sport; however, QoL was not significantly improved by participation in social activities in general. Manlhiot^26^ demonstrated that children with CHDs who have healthy siblings have reduced QoL, suggesting that affected children may compare their physical abilities unfavourably with a ‘normal’ sibling. Interestingly, affected children in our cohort who participated regularly in sport scored higher on psychosocial functioning than children who did not, although there was no significant difference in self-reported physical functioning scores. Several researchers have also shown that regular involvement in sports or vigorous recreational physical activity benefits children's well-being and reduces emotional and behavioural problems.^32^ ^33^ It is clear that participation in sporting or social activities represents a complex mediator of risk, influenced partly by the physical limitation imposed by a CHD. It is conceivable that some children with CHDs who participate fully in school and sports rate their psychosocial QoL high despite scoring their physical functioning lower, because they understand implicitly that they are ‘successfully’ negotiating the physical limitations of their condition.

Our study explores the impact of living with a CHD for children who have a range of defects, many of which might be considered surgically ‘corrected’ in infancy. Crucially, the mediating factors that might protect children from adverse QoL outcomes are likely to differ between individuals and may change over time.^9^ Experiencing recent or frequent health interventions may increase awareness of CHD as an ongoing health burden, with a consequent negative impact on QoL. We found decreased QoL associated with regular medication or cardiac interventions; in contrast, diagnostic severity was not an independent predictor of QoL. Greater attention may therefore need to be paid to the cumulative burden of interventions and medical care experienced by young patients.

PROMs are a key development as many healthcare outcomes, such as reduced symptoms or improvements in functional status and QoL, can only be assessed by patients.^7^ PROMS are increasingly being used to support shared decision-making between patients and clinicians.^34^ We have demonstrated the feasibility of using the PedsQL to obtain patient-reported outcomes for UK children living with a CHD. The added benefit of using measures designed specifically to capture the child's perspective has been clearly highlighted.^35^ Paediatric patient-report measures should be considered for integration into routine monitoring of chronic childhood disorders, and specifically to enrich cardiac audit and provide an additional source for evaluating and effecting improvements in care. A child-centred approach is fundamental to communication between children, families, health and education professionals about individual care, as well as to promoting good coping strategies and social inclusion to enhance the lives of children with CHDs for whom long-term survival in adulthood is now a realistic expectation.

## Supplementary Material

Web supplement
