# Supplementary material for: Patient-reported quality of life outcomes for children with serious congenital heart defects
Source: Arch Dis Child. 2014 Jan 9;99(5):413–9. doi: 10.1136/archdischild-2013-305130 (PMC3995241; doi:10.1136/archdischild-2013-305130)
Supplement: Web supplement [file archdischild-2013-305130-s1.pdf]

Supplementary Table 1: Characteristics of children in the UKCSCHD who participated in the quality of life outcomes study

	Children with CHDs who completed questionnaires N = 477		UKCSCHD cohort survivors who did not complete questionnaires N = 2486	
	N (%)	Missing [N (%)]	N (%)	Missing [N (%)]
**Sex**		0		53 (2%)
- Male	268 (56%)		1381(56%)	
**Year of birth**		0		0
- 1992	32 (7%)		255 (10%)	
- 1993	154 (32%)		830 (33%)	
- 1994	147 (31%)		787 (32%)	
- 1995	144 (30%)		614 (25%)	
**Cardiac Prognostic Severity (CPS)†**		1 (<1%)		9 (<1%)
- Curative	102 (21%)		670 (27%)	
- Corrective	271 (57%)		1399 (56%)	
- Palliative	103 (22%)		408 (16%)**	
**Primary cardiac diagnosis‡**		0		0
- Hypoplastic left heart / MA	9 (2%)		37 (1%)	
- Tricuspid atresia	7 (1%)		34 (1%)	
- Double inlet ventricle	13 (3%)		41 (2%)	
- Pulmonary atresia + IVS	14 (3%)		43 (2%)	
- Pulmonary atresia + VSD	23 (5%)		72 (3%)	
- Truncus arteriosus	16 (3%)		37 (1%)	
- Complete AVSD	37 (8%)		255 (10%)	
- TGA	104 (22%)		394 (16%)	
- Tetralogy of Fallot	41 (9%)		254 (10%)	
- TAPVC	16 (3%)		97 (4%)	
- VSD	83 (17%)		585 (24%)	
- Aortic stenosis	18 (4%)		66 (3%)	
- Pulmonary stenosis	32 (7%)		142 (6%)	
- Coarctation of the aorta	53 (11%)		309 (12%)	
- Miscellaneous*	16 (3%)		120 (5%)	
**Number of cardiac interventions#**		0		0
- Median (IQR)	2 (1, 2)		1 (1, 2)	

Notes: *Includes congenitally corrected transposition of the great arteries, partial atrioventricular septal defect, aortopulmonary window, aortic stenosis, atrial septal defect, and rare complex defects affected less than 20 children within the original UKCSCHD cohort; **includes 5 transplants; # median number of interventions (IQR=interquartile range) – as recorded in hospital records.

‡ **Primary cardiac diagnosis** was a hierarchical classification adapted from Wren[14], The methodology for assigning primary diagnoses to 1,768 children with multiple defects was validated independently by three raters (RK, CB, CW). Based on cardiac diagnoses in medical records, children assigned a primary diagnosis were 1,738 (98%), 1,610 (91%) and 1,146 (65%) for each rater; this increased to 1,761 (99.7%), 1,689 (95.5%) and 1,658 (93.8%) respectively using records of surgical procedures (Interrater agreement: κ=0.83). A 'miscellaneous' category included defects found in fewer than 40 children: congenitally corrected transposition of the great arteries (n=24), partial atrioventricular septal defect (n=20), aortopulmonary window (n=26), atrial septal defect (n=36) and rarer diagnoses.

† **Cardiac Prognostic Severity (CPS Score)** score adapted from Lane[15]**.** CPS groups were: no intervention-children who received no surgical intervention; curative-children who had successful repair of atrial or ventricular septal defect, pulmonary stenosis or total anomalous pulmonary veins and had no additional cardiac defects; corrective-children who had a procedure which approximated normal anatomy and restored biventricular function, with no expectation of future surgery during childhood; palliative-children whose surgery did not restore biventricular function, including children for whom multi-stage repair was only partially achieved, only a single functional ventricle circulation was possible, who had a valve replacement which required later revision or who had a cardiac transplant.

Supplementary Table 2: Univariable regression models to explore factors associated with PedsQL outcome scores

PedsQL™	Summary score			Physical functioning			Psychosocial functioning		
	Est.	SE	*P*	Est.	SE	*P*	Est.	SE	*P*
**Individual factors**									
Presence of CHD	**-3.25**	**0.62**	**<0.001**	**-1.39**	**0.39**	**<0.001**	**-4.10**	**0.73**	**<0.001**
Female	-0.14	0.55	0.79	-0.41	0.37	0.26	0.22	0.66	0.74
Age (years)	-0.32	0.28	0.26	-0.22	0.18	0.21	-0.35	0.33	0.29
White Ethnicity	0.09	1.00	0.93	0.48	0.67	0.47	0.02	1.19	0.98
Birthweight (per kg)	0.52	0.43	0.23	0.21	0.35	0.56	0.78	0.53	0.13
**Parent† & family factors**									
Parental education level									
- None/GCSE only	*ref.*			*ref.*			*ref.*		
- A level	-0.69	0.70	0.32	-0.25	0.47	0.59	-0.92	0.83	0.27
- Degree	-0.12	0.65	0.86	-0.10	0.43	0.82	-0.11	0.78	0.89
Number of full-time equivalent working parents†	**1.40**	**0.52**	**<0.001**	0.13	0.35	0.71	**1.71**	**0.61**	**<0.001**
Number of siblings at home	-0.39	0.31	0.21	-0.10	0.20	0.61	-0.38	0.38	0.31
Two parents† at home now	**1.52**	**0.68**	**0.03**	0.39	0.45	0.39	**1.95**	**0.83**	**0.02**
Two parents† at birth	2.05	1.50	0.17	0.71	0.95	0.46	2.82	1.77	0.11
Mother's age at birth	0.08	0.06	0.16	0.01	0.04	0.87	0.10	0.07	0.16
Father's age at birth	0.03	0.05	0.52	0.02	0.03	0.52	0.04	0.06	0.49
**Co-morbidities**									
Presence of non-cardiac longstanding illness									
- None	*ref.*			*ref.*			*ref.*		
- Yes, not limiting	**-2.46**	**0.97**	**0.01**	**-1.21**	**0.60**	**0.04**	**-2.90**	**1.15**	**0.01**
- Yes, limiting	**-8.85**	**1.11**	**<0.001**	**-4.40**	**0.69**	**<0.001**	**-10.0**	**1.29**	**<0.001**
Regular non-cardiac medications	**-5.30**	**0.76**	**<0.001**	**-2.50**	**0.54**	**<0.001**	**-5.66**	**0.86**	**<0.001**
Problems with vision	**-2.89**	**0.64**	**<0.001**	**-1.01**	**0.42**	**0.02**	**-3.34**	**0.76**	**<0.001**
Problems with hearing	**-2.55**	**0.72**	**<0.001**	-0.71	0.48	0.14	**-3.18**	**0.87**	**<0.001**
Problems with speech	**-7.79**	**1.11**	**<0.001**	**-3.07**	**0.78**	**<0.001**	**-9.57**	**1.40**	**<0.001**
**School & Daily Life Activities**									
Type of schooling									
- Mainstream school	*ref*			*ref*			*ref*		
- Mainstream with assistance	**-6.33**	**0.96**	**<0.001**	**-1.71**	**0.58**	**0.003**	**-8.52**	**1.19**	**<0.001**
- Special school /unit	**-11.8**	**1.74**	**<0.001**	**-4.35**	**1.10**	**<0.001**	**-15.2**	**2.07**	**<0.001**
School absence in last year									
- Never	*ref.*			*ref.*			*ref.*		
- < 1 week	**-1.52**	**0.69**	**0.03**	**-0.92**	**0.44**	**0.03**	**-1.80**	**0.83**	**0.03**
- 1-2 weeks	**-3.60**	**0.99**	**<0.001**	**-1.86**	**0.62**	**0.003**	**-4.42**	**1.13**	**<0.001**
- 2 weeks -1 month	**-7.19**	**1.28**	**<0.001**	**-2.47**	**0.88**	**0.004**	**-8.42**	**1.45**	**<0.001**
- >1 month	**-10.9**	**2.02**	**<0.001**	**-3.65**	**1.28**	**0.005**	**-13.5**	**2.22**	**<0.001**
Frequency of sport*	**1.11**	**0.21**	**<0.001**	**0.41**	**0.13**	**0.002**	**1.40**	**0.25**	**<0.001**
Frequency of social activities**	**0.50**	**0.11**	**<0.001**	**0.21**	**0.07**	**<0.001**	**0.66**	**0.14**	**<0.001**
**Cardiac factors**									
Cardiac Prognostic Severity (CPS)									
- No cardiac disorder	*ref.*			*ref.*			*ref.*		
- Curative	**-2.73**	**0.98**	**<0.001**	-0.62	0.63	0.33	**-3.59**	**1.16**	**<0.001**
- Corrective	**-3.10**	**0.71**	**<0.001**	**-1.44**	**0.45**	**<0.001**	**-3.89**	**0.83**	**<0.001**
- Palliative	**-4.90**	**1.12**	**<0.001**	**-2.55**	**0.73**	**<0.001**	**-5.76**	**1.28**	**<0.001**
Number of cardiac interventions	**-0.95**	**0.11**	**<0.001**	**-0.65**	**0.13**	**<0.001**	**-0.97**	**0.12**	**<0.001**

Key: Est. (estimate); SE (standard error); *Number of occasions child takes part in a sporting activity (not school PE) per week; **Number of occasions outside of school hours that a child takes part in sport, plays with friends, or attends non-sport clubs per week; †Includes carers/guardians;
